# Post-acquisition standardization of positron emission tomography images

**DOI:** 10.3389/fnume.2023.1210931

**Published:** 2023-09-12

**Authors:** Aliasghar Mortazi, Jayaram K. Udupa, Dewey Odhner, Yubing Tong, Drew A. Torigian

**Affiliations:** Medical Image Processing Group, Department of Radiology, University of Pennsylvania, Philadelphia, PA, United States

**Keywords:** positron emission tomography, standardized uptake value, PET standardization, SUV variability, tumor quantification

## Abstract

**Purpose:**

Tissue radiotracer activity measured from positron emission tomography (PET) images is an important biomarker that is clinically utilized for diagnosis, staging, prognostication, and treatment response assessment in patients with cancer and other clinical disorders. Using PET image values to define a normal range of metabolic activity for quantification purposes is challenging due to variations in patient-related factors and technical factors. Although the formulation of standardized uptake value (SUV) has compensated for some of these variabilities, significant non-standardness still persists. We propose an image processing method to substantially mitigate these variabilities.

**Methods:**

The standardization method is similar for activity concentration (AC) PET and SUV PET images, with some differences, and consists of two steps. The *calibration step* is performed only once for both AC PET or SUV PET, employs a set of images of normal subjects, and requires a reference object, while the *transformation step* is executed for each patient image to be standardized. In the calibration step, a standardized scale is determined along with 3 key image intensity landmarks defined on it: the minimum percentile intensity *s*_min_, median intensity *s_m_*, and high percentile intensity *s*_max_. *s*_min_ and *s_m_* are estimated based on image intensities within the body region in the normal calibration image set. The optimal value of the maximum percentile *β* corresponding to the intensity *s*_max_ is estimated via an optimization process by using the reference object to optimally separate the highly variable high uptake values from the normal uptake intensities. In the *transformation step*, the first two landmarks—the minimum percentile intensity *p_α_*(*I*), and the median intensity *p_m_*(*I*)—are found for the given image *I* for the body region, and the high percentile intensity *p_β_*(*I*) is determined corresponding to the optimally estimated high percentile value *β*. Subsequently, intensities of *I* are mapped to the standard scale piecewise linearly for different segments. We employ three strategies for evaluation and comparison with other standardization methods: (i) comparing coefficient of variation (CV*_O_*) of mean intensity within test objects *O* across different normal test subjects before and after standardization, (ii) comparing mean absolute difference (MD*_O_*) of mean intensity within test objects *O* across different subjects in repeat scans before and after standardization, and (iii) comparing CV*_O_* of mean intensity across different normal subjects before and after standardization where the scans came from different brands of scanners.

**Results:**

Our data set consisted of 84 FDG-PET/CT scans of the body torso including 38 normal subjects and two repeat-scans of 23 patients. We utilized one of two objects—liver and spleen—as a reference object and the other for testing. The proposed standardization method reduced CV*_O_* and MD*_O_* by a factor of 3–8 in comparison to other standardization methods and no standardization. Upon standardization by our method, the image intensities (both for AC and SUV) from two different brands of scanners become statistically indistinguishable, while without standardization, they differ significantly and by a factor of 3–9.

**Conclusions:**

The proposed method is automatic, outperforms current standardization methods, and effectively overcomes the residual variation left over in SUV and inter-scanner variations.

## 1. Introduction

### 1.1. Background and rationale

Cancer is the second most common cause of death in the United States and is a significant health problem worldwide. In 2019, about 1.8 million new cancer cases and about 0.6 million cancer deaths were reported in the United States (1). Positron emission tomography (PET), a non-invasive molecular imaging technique, is one of the major clinical imaging modalities used routinely for comprehensive body-wide diagnostic assessment of patients with cancer and other non-cancerous disorders. PET detects, measures, and localizes gamma rays emitted from annihilation events between positrons (emitted by administered positron-emitting isotopes) and electrons, providing a method to distinguish tissues that have differential radiotracer activities. For example, abnormal changes in tissue metabolic activity can be detected with ^18^F-fluorodeoxyglucose (FDG)-PET imaging before structural changes are detectable with computed tomography (CT) or magnetic resonance imaging (MRI). As such, metabolic activity measured from FDG-PET is an important biomarker that is clinically utilized for diagnostic, staging, prognostication, and treatment response assessment purposes in patients with cancer (2–5).

Although qualitative assessment of PET images in clinical practice is routinely performed, quantitative assessment is encouraged to decrease inter-reader variability and to improve diagnostic performance of study interpretation. In early attempts for disease quantitative assessment in PET images, the percent of administered dose per gram of tissue was used as a measure of tumor uptake (6). However, after comparing this metric among different patients, it was discovered that this value is affected by the patient size as well as by the radiotracer dose administered. To compensate for these factors, another quantitative measurement was introduced called *Standardized Uptake Value* (SUV), which is the decay-corrected tissue activity concentration of radiotracer in a region of interest (ROI) divided by the injected radiotracer dose per unit body weight (or alternatively body surface area or lean body mass) (see Equation 1) (7, 8). SUV measurement has been widely utilized for semi-quantitative PET assessment in clinical practice given its ease of use. For any PET image *I*, the value *I*(*v*) at any voxel *v* represents the activity concentration (AC) of the radiotracer (in units of MBq/ml). This value is converted to SUV(*v*) at *v* by using the formula:

(1) $$\mathrm{SUV}(v)=\frac{I(v)}{\mathrm{injected}\;\mathrm{radiotracer}\;\mathrm{dose}/\mathrm{body}\;\mathrm{weight}}.$$

Note that injected radiotracer dose is in units of MBq, and that body weight is in units of *g*, where it is assumed that the average mass density of the human body is 1 g/ml [such that 1 g = 1 ml and SUV(*v*) is therefore unitless]. The factors that can adversely affect the accurate and precise measurement of tissue radiotracer uptake as portrayed in PET images can be divided into two categories: patient-related factors and technical factors. Patient-related factors include differences in body weight, body composition, body habitus, serum glucose levels, etc. Technical factors include differences in radiotracer uptake period, partial volume effects, the size and placement of the region of interest (ROI), image acquisition methods, attenuation correction methods, image reconstruction methods, etc. (3, 9–11). SUV only partially compensates for certain factors such as patient body weight and administered radiotracer dose.

The uncompensated factors can make accurate and reproducible disease quantification via PET acquisitions very challenging, potentially leading to diagnostic errors during disease staging and response assessment that may adversely affect patient management and outcome, not to mention site-to-site variations and their attendant issues. Equally importantly, these factors cause non-standardness of SUV numerically and pose challenges to image processing and analysis methods. Even if it were possible to segment object/pathology automatically with advanced deep learning methods with the presence of non-standardness, disease measurement within segmented entities using SUVs will vary substantially. Needless to say, the original raw PET images from which voxel-wise SUV is estimated also pose challenges of at least similar magnitude. As such, methods have been developed to compensate for some of these uncompensated factors.

### 1.2. Related works

Some methods operate at the *image acquisition level* such as by using a phantom, by modifying image reconstruction, or by standardizing the parameters of scan data acquisition. For example, Bae et al. performed standardization of PET/CT scanners using phantom tests in a multicenter phase 2 trial of patients with peripheral T-cell lymphoma (12). Namias et al. used a simple cylindrical phantom based on resolution and noise measurements for harmonization of PET/CT images (13). Ferretti et al. investigated a method using a single reconstruction data set together with a post-reconstruction algorithm for SUV harmonization to address the problems of exaggerated SUV due to point-spread function (PSF) corrections in PET/CT reconstruction (14).

Other methods operate at the *patient level* by controlling or correcting for the amount of radiotracer dose administered in the setting of radiotracer extravasation, compensating for the patient's serum glucose level, or by controlling the allowable delay time for radiotracer uptake. For example, Jahromi et al. compared the accuracy of SUV corrected by serum glucose levels (SUV_gluc_) to 4 other commonly used semi-quantitative metrics for evaluation of pulmonary nodules on FDG-PET scans and concluded that SUV_gluc_ was the most accurate SUV parameter (15). Laffron et al. proposed a simple method derived from kinetic model analysis to normalize decay-corrected SUV for injection-to-acquisition time differences within the range of 55–110 min in FDG-PET imaging (16).

In yet other methods, standardization is performed at the *image post-processing stage* by using various methods such as digital PET phantoms, anatomical standardization with *Z*-scores, or various image transformation methods. For example, Hara et al. proposed the use of anatomical standardization of PET images of the torso region via construction of a normal torso model and subsequent determination of the SUV scores as *Z*-score indices for measuring the abnormalities in an FDG-PET scan image (17). Scarpelli et al. identified the optimal transformation for producing normal distributions of tumor SUVs on pre-treatment and post-treatment FDG-PET and ^18^F-fluorothymidine (FLT)-PET images by iterating the Box-Cox transformation parameter and selecting the parameter that maximized the Shapiro–Wilk *P*-value (18). Orlhac et al. proposed the use of a harmonization method (ComBat) initially described for genomic data to normalize radiomic features as measured in PET for removing the center effect while retaining pathophysiologic information (19).

Image-acquisition-level approaches are not very practical and cannot be used to analyze data sets that have been acquired without following the regimen required by them. Patient-level approaches do not fully correct for the non-standardness of SUV, as there is often still variability in radiotracer uptake and since serum glucose level differentially affects FDG uptake within different tissue types, leading to overcorrections and under-corrections of SUV. Post-acquisition methods such as *Z*-scores generally perform a linear correction and do not account for non-linear variations that often exist among data sets obtained from different patients. Also, most of these methods perform harmonization for a specific organ and cannot be applied to the whole-body PET images or to other organs without requiring major modifications. Moreover, they require the organ of interest to be segmented in order to normalize. Furthermore, a major drawback of current PET standardization/harmonization methods is the lack of appropriate and logical quantitative methods and metrics for evaluation. The goals of this paper are not only to demonstrate post-acquisition techniques to standardize activity concentration (AC) PET images as well as SUV PET images but also to address the evaluation problems. We show how the proposed standardization techniques substantially improve tissue-specific meaning across patients upon standardization and also how the new metrics enable us to measure and compare among different standardization/normalization methods.

Standardization has been studied extensively for magnetic resonance imaging (MRI) starting with the method introduced by Nyul et al.^1^ (20, 21). They proposed a 2-step process consisting of *calibration* and *transformation*. In the calibration step, landmarks in the image intensity space (such as mean, median, quartiles, and deciles) derived from image histograms of the foreground of the image are found on a set of images for creating an intensity mapping model. In the intensity transformation step, the intensities of any given patient image are non-linearly mapped by using the landmarks to guide the transformation. One aspect of the MRI intensity standardization challenge that has direct relevance to AC PET and SUV PET images is the strategy to handle high outlier intensities. In MRI, these intensities have been shown to be due to noise and artifacts and have a similar behavior among the most commonly used MRI sequences (20). In PET, particularly FDG-PET, which is the focus of this paper, they arise due to noise *as well as* the large dynamic range of high FDG concentrations in pathologic tissue regions and in some normal organs. In MRI image analysis, the positive influence of intensity standardization on other image operations such as non-uniformity correction (22), segmentation (23), registration (24), and even standardization itself has been demonstrated (22). In PET image analysis, such avenues have yet to be explored.

In this paper, we propose a new standardization method for AC PET and SUV PET images inspired by the MRI standardization techniques of Nyul et al. Although the proposed techniques have similarities to the approach of Nyul et al., a direct application of that approach to PET/SUV images will not work, as we demonstrate in Section 3, for three key reasons: (i) The outlier intensities in MRI are better behaved than the high intensities in PET/SUV images, always lying at or beyond the 99.8 percentile level (20), independent of the MRI pulse sequence protocol. They are much harder to handle in PET/SUV images; (ii) In MRI, the outlier intensities and intensities due to pathology do not confound, as such calibration for standardization can be performed directly on the patient images irrespective of whether they are normal or abnormal. In PET/SUV images, this is not the case, and calibration must be performed based on normal scans. Furthermore, the cut off percentile is to be determined in a reference-organ-specific manner via an optimization process, as demonstrated in this paper; and (iii) In view of (ii), PET standardization, unlike MRI, requires a reference organ whose normal uptake is low enough that it is not mixed up with extremely variable high-uptake regions. High-uptake organs like heart, kidneys, and bladder are thus not useful as reference organs for PET standardization.

Despite efforts to control specific patient-related and technical factors, current PET images, including derived SUV measurements with an implicit standardization, still have considerable variability across subjects in similar tissue regions that are normal. Therefore, the goal of the standardization method is to reduce this variability from the overall effect of multiple variables, without focusing on any specific variables individually, under the assumption that we expect PET image values to be similar in comparable normal tissue regions in different subjects. This goal is in line with that of societies and groups such as the SNMMI (Society of Nuclear Medicine and Molecular Imaging), EANM (European Association of Nuclear Medicine), American College of Radiology (ACR), Radiological Society of North America (RSNA), Quantitative Imaging Biomarkers Alliance (QIBA), and Quantitative Imaging Network (QIN) of the National Cancer Institute (NCI) as part of the broader effort to improve the accuracy and reproducibility of quantitative PET imaging, as the proposed standardization method would improve the subsequent measurement of whatever quantitative metrics of interest are sought after (25–28).

Our proposed methods do not assume that hepatic or splenic metabolism is exactly the same across normal subjects. However, they do assume that normal hepatic or splenic metabolism should be within an expected range of variability amongst a population of subjects. Such an assumption is made all the time in the application of many types of diagnostic tests when reporting what is “normal” and “abnormal” in terms of the test results, which is largely based on our knowledge of human physiology, technical performance of the particular diagnostic test at hand, and observations of organ behaviors during PET scan interpretation.

Understanding what is “normal” is critically important to the detection, quantification, and diagnostic interpretation of PET images, as it allows one to (1) detect abnormality when present, even if subtle or diffuse within an organ of interest, given that once “normal” has been defined, everything that is “outside” normal can be defined as “abnormal”; (2) enable quantification of subtle disease and even inconspicuous disease when present beyond what is due to normal radiotracer uptake; and (3) improve accuracy of lesion-to-background measurements, which is important for quantitative assessments in cancer and in non-cancer related disorders.

Although PET scans reflect absolute measures of radiotracer uptake at the time of imaging as well as variations in imaging technique and human biological status, there is no reason to ignore information gleaned from use of populations of studies in terms of the normal level and range of radiotracer uptake within individual organs and from knowledge of human organ physiology in order to facilitate detection and quantification of pathology whenever present.

Our approach for both AC PET and SUV PET images, as described in Section 2, consists of a one-time calibration step, wherein the parameters of the standardization mapping are determined (learned), followed by the transformation step performed on any acquired patient image. Calibration is carried out by using only normal (or near-normal) images and separately for AC PET and SUV PET images, and the transformation step is applied to any given image—normal or abnormal. Section 2 also describes our strategies for evaluating the effectiveness of standardization. In Section 3, we present detailed results in comparison to direct application of the MRI standardization approach and other standardization strategies. We state our concluding remarks in Section 4.

An early version of this work was presented at the SPIE Medical Imaging Conference held in Houston in February 2020 whose proceedings contained the abbreviated paper. The present paper differs from the conference paper in major ways: (i) It fully describes the background and rationale with a comprehensive review of the literature which was lacking in the conference paper; (ii) It gives full details of the method and all associated algorithms while the conference paper included just an outline for just the AC PET images and did not include SUV standardization; and (iii) The evaluation is significantly expanded in this paper over the conference paper to include both AC PET and SUV PET images, comparative analysis with other methods, and repeat scan data sets of patients to show the reproducibility of the method.

## 2. Materials and methods

### 2.1. Overview and notations

Let I be a set of 3D PET images of a body region B, comprised of a stack of sequential transverse slices. In this paper, we will be studying standardization of both AC PET and SUV PET images. The standardization process is mostly the same for both AC PET and SUV PET images. Thus, we may think of I as representing either a set of AC PET images or a set of SUV PET images. Our description will be general without referring to AC or SUV PET images specifically, except when there is a deviation in the process between them, in which case, the differences will be explained.

For any image *I* in I, we will denote its standardized image by *I_s_*. We will denote the entire standardization mapping by *ψ*. Thus, per our notation, for any image *I* in I, *I_s_* = *ψ*(*I*). Our standardization strategy employs certain landmarks or special features of interest in the image intensity or voxel value space, observable on image intensity distributions or histograms, defined as follows. For any image *I* in I, we will denote its minimum and maximum intensities by min(*I*) and max(*I*), and three special percentile values (more about this later), called lower percentile value, median value (50th percentile), and upper percentile value by *p_α_*(*I*), *p_m_*(*I*), and *p_β_*(*I*), respectively. Here *α* and *β* denote the lower and upper percentiles; for example, we may have *α* = 5 denoting the 5th percentile in *I* and *β* = 95 denoting the 95th percentile in *I*, and correspondingly, the actual image intensity values corresponding to these percentiles may be *p_α_*(*I*) = 52 and *p_β_*(*I*) = 3,007.

The proposed standardization method consists of two main steps: calibration and transformation. The calibration step is performed only once for a scanner or set up while the transformation step is executed for each acquired patient image. In the *calibration* step, a standardized scale is determined along with key image intensity landmarks defined on it, named *s*_min_, *s_m_*, and *s*_max_ by using a subset I*_c_* of I. Set I*_c_* is used expressly for calibration purposes only and the images in this set are assumed to be normal^2^. The idea is that *s*_min_, *s_m_*, and *s*_max_ have a meaning similar to *p_α_*(*I*), *p_m_*(*I*), and *p_β_*(*I*), respectively, except that they denote statistical average locations (in the intensity space) of the latter obtained from the images in I*_c_*. In the *transformation* step, for any given image *I* to be standardized, where $I\;\in \;I_{t}\;=\;I\;-\;I_{c}$ is not necessarily normal, the same landmarks are determined in *I*, the mapping that results when the landmarks of *I* are matched to the landmarks on the standardized scale is computed, and *I*'s voxel intensities *I*(*v*) are transformed to *I_s_*(*v*) according to the mapping. Landmarks *p_m_*(*I*) and *p_β_*(*I*) play key but different roles in standardization. *p_m_*(*I*) allows shifting the overall intensity in *I* to a reference value. *p_β_*(*I*) helps in finding that reference reliably. The main reason for choosing *p_β_*(*I*) ≠ max(*I*) is that the upper tail of the histogram of *I* is affected by artifacts, outlier intensities, and very high uptake values due to the presence of pathological conditions and other high-level accumulations of radiotracers which cause significant variation among subjects and scanners. As we show in this paper, such variations in PET images can lead to undesired SUV variations among healthy organs from different subjects and scanners. Following the idea introduced by Nyul et al. (20, 21), to solve this problem, we use *p_α_*(*I*) and *p_β_*(*I*) as landmarks such that only within the interval [*p_α_*(*I*), *p_β_*(*I*)] do we seek to uniformize^3^ intensity meaning across subjects. Finally, intensities in [min(*I*), *p_α_*(*I*)] are transformed by using (extrapolating) the mapping associated with [*p_α_*(*I*), *p_m_*(*I*)]. Similarly, intensities in [*p_β_*(*I*), max(*I*)] are transformed by using the mapping associated with [*p_m_*(*I*), *p_β_*(*I*)].

In the following sections, we first explain the calibration and transformation steps and then describe our evaluation strategy together with a brief outline of two common methods from the literature with which we have compared our standardization method.

### 2.2. Calibration

**Figure 1:**
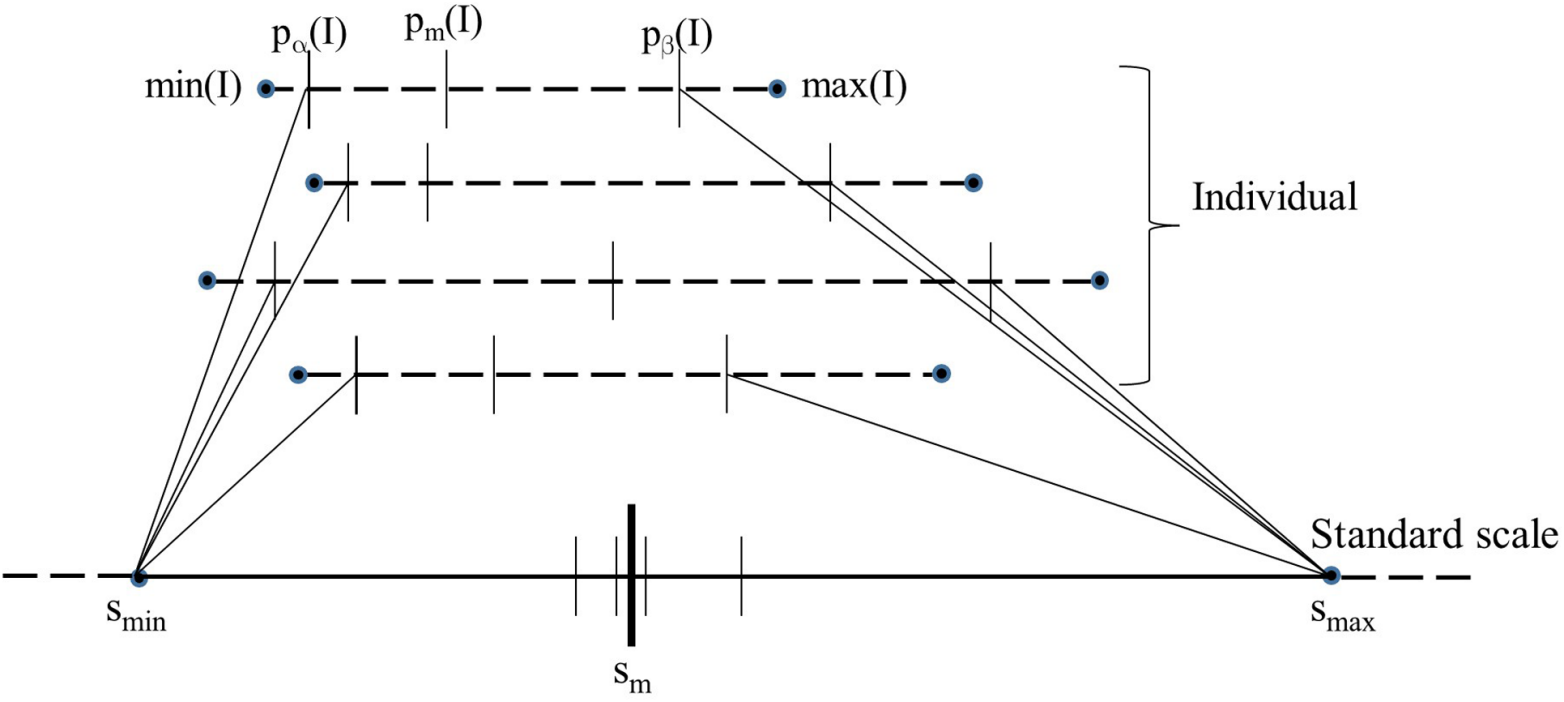
Schematic illustration of the calibration process. The image intensity scales of 4 images are shown on the top as dotted lines. The 5 key landmarks of each image are also marked on the intensity scale. The standard scale is shown at the bottom as a solid line. The position of *p_m_*(*I*) when each of the 4 images is mapped linearly from their respective [*p_α_*(*I*), *p_β_*(*I*) to [*s*_min_, *s*_max_] of the standard scale is depicted on the standard scale as small vertical.

Figure 1 is a schematic depiction of the calibration process. Given the set I*_c_* of images of normal subjects, the calibration process outputs the standard scale along with its parameters, namely the landmark locations for *s*_min_, *s_m_*, and *s*_max_ on the standard scale. The process consists of three steps: (i) Defining a standard scale, (ii) identifying landmarks on individual image scales, and (iii) determining landmarks on the standard scale. We emphasize again that the calibration process uses only *normal* images as explained above.

#### 2.2.1. Defining a standard scale

The length of the standard scale defined by the interval [*s*_min_, *s*_max_] is chosen such that we do not lose “resolution” of SUVs contained in the input image (irrespective of whether the input is an AC PET image or a SUV PET image) as it undergoes standardization mapping. Since min(*I*) is typically 0 for PET images, we select *s*_min_ = 0 for both AC PET and SUV PET standardization. *s*_max_ is chosen so that no two distinct SUVs in the input image that are clinically meaningful map to the same SUV after standardization. See Section 3 for further details. Recall that *s*_max_ denotes roughly the maximum SUV on the standard scale for the normal portion of the activity (determined from normal images used for calibration) and not the actual maximum possible SUV in any patient image.

#### 2.2.2. Identifying landmarks on individual image scales

Among the 5 landmarks (see Figure 1), the 1st and 5th landmarks min(*I*) and max(*I*) are selected to be the actual minimum and maximum voxel value in *I*, respectively. The second landmark *p_α_*(*I*) is set equal to min(*I*) which is typically 0 in our images. The remaining two parameters, *p_m_*(*I*) and *p_β_*(*I*), are selected based on the histogram of *I* as follows.

**Figure 2:**
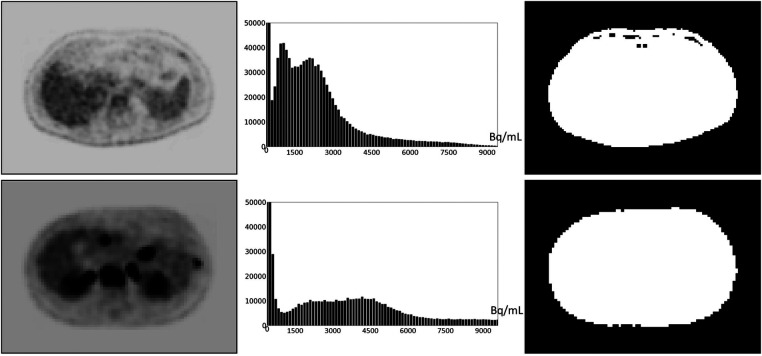
An FDG-PET axial image slice at the mid abdominal level selected from a 3D body torso image *I* (left), the histogram of the full 3D image *I* (middle), and the binary image (right) resulting from thresholding *I* at mean(*I*). (Top): Image from a normal subject. (Bottom): Image from a patient with metastatic cancer. For better visualization, some background regions are trimmed in the slice displays on the left.

Defining and estimating *p_m_*(*I*): Based on our examination of body-wide FDG-PET/CT scans of 552 patients, the histogram of the full 3D AC PET and SUV PET images is typically bimodal. The first mode is situated close to 0 and corresponds to activity in the background of the image outside the body region, and the second mode represents the body region. Figure 2 displays the histograms of the full body torso 3D SUV PET image from FDG-PET/CT acquisitions of one normal subject and one cancer patient. A PET axial slice at the mid abdominal level is also displayed in the figure. We select *p_m_*(*I*) to be the median value within the body region (second mode) in *I*. To find the body region, we threshold *I* at the mean, denoted by mean(*I*), of the image intensity values over the whole volume of *I*. The thresholded results are also shown in Figure 2 for the two studies. This simple technique worked well as verified on all 552 images tested. Note that perfect segmentation of the body region is not needed here since *p_m_*(*I*) is the median value within the segmented region and is not affected by minor imprecisions in the thresholded outcome. To verify our assertion, we segmented the body region accurately in all data sets in I*_c_* by thresholding at the volume mean followed by a filling operation and performing manual corrections as needed. We found the mean ± SD (standard deviation) of the difference in the median values estimated by the two methods of segmentation in PET images over all data sets in I*_c_* to be 0.667 ± 0.925.

Defining and estimating *p_β_*(*I*): The landmark *p_β_*(*I*) corresponding to the upper percentile value is the most crucial from the standardization perspective and the most challenging to define, considering the large and variable dynamic range of the image values, the highly variable high uptake values, and the variability within normal tissues that we need to handle. To exemplify, the AC scale among our data sets of normal subjects in I*_c_* varies from [700, 10,200] to [4,600, 56,100] Bq/ml and the corresponding SUV scale varies from [0.2, 1.6] to [9.6, 13.25]. Also, the mean value scale over healthy liver and spleen among AC data set varies from 3,050 to 18,659 Bq/ml and the corresponding SUV scale varies from 0.66 to 3.33. These variabilities pose challenges for quantitative analysis as well as for 3D visualization such as via maximum intensity projection in a consistent manner. Our idea is to choose a *reference organ O* and select *β* to be the largest percentile such that, upon standardizing each *I* in I*_c_* by using *p_β_*(*I*), the coefficient of variation over the samples in I*_c_* of the standardized mean intensity within *O* is the smallest.

To elaborate, let *μ_O_*(*I_s_*) denote the mean intensity value of a specific image *I* in $I_{c}$ within *O* after *I* is standardized to *I_s_* by using a given upper percentile *b* and the corresponding upper percentile landmark *p_b_*(*I*), let *m_O_*(*b*) and *σ_O_*(*b*) denote the mean and standard deviation of *μ_O_*(*I_s_*) over all images in $I_{c}$, and let *δ_O_*(*b*) be the coefficient of variation of *μ_O_*(*I_s_*) over images in $I_{c}$:

(2) $$\delta_{O}(b)=\frac{\sigma_{O}(b)}{m_{O}(b)}.$$

Then the optimum upper percentile *β* is chosen to be that *b* which minimizes *δ_O_*(*b*) over all upper percentile values over a certain interval [*b_L_*, *b_H_*]:

(3) $$\beta =\begin{array}{c} \arg\;\min\\ b \end{array}\{\delta_{O}(b):\;b_{L}\leq b\leq b_{H}\}.$$

We have taken [*b_L_*, *b_H_*] = [90, 100]. The liver is commonly used as a reference organ in FDG-PET. For example, it is used as a reference organ in the PET response criteria in solid tumors (PERCIST) response assessment system because it is relatively stable and uniform in terms of FDG uptake from scan to scan, is well-defined and sufficiently large, and has more FDG-uptake than other background organs such as adipose tissue or lung so that it is easily visible and measurable (29). Other more FDG-avid organs like brain and heart have a lot more variable FDG uptake between scans and have more heterogeneous FDG uptake within the organs themselves. The spleen is more variable in terms of FDG uptake compared to liver, but still generally has uniform uptake and can also be used as a reference organ. Therefore, in this work, we have used both liver and spleen as reference organs for estimating *β* for the calibration process. As we will demonstrate in Section 3, *β* estimated by using the two organs as reference yields the same value. We note that these organs are needed as reference only in the calibration step and not for performing standardization transform on a patient scan.

#### 2.2.3. Determining landmarks on the standard scale

Parameters *s*_min_ and *s*_max_ of the standard scale are determined as explained above. To estimate *s_m_*, first the intensities in [*p_α_*(*I*), *p_β_*(*I*)] in the images *I* in $I_{c}$ are mapped linearly to the interval [*s*_min_, *s*_max_] on the standard scale; see Figure 1. Denoting this linear mapping by λ(x), *s_m_* is defined as the mean of the mapped values $\lambda (\;p_{m}(I))$ on the standard scale over all I in $I_{c}$:

(4) $$s_{m}=\frac{1}{\left|I_{c}\right|}\sum_{I\in I_{c}}\lambda (\;p_{m}(I)),$$

where |$I_{c}$| denotes the cardinality of $I_{c}$. In Figure 1, $\lambda (\;p_{m}(I))$ values for 4 images are illustrated. Note how *p_m_*(*I*) and *p_β_*(*I*) both play an important role in defining *s_m_* and *s*_max_. Note also that the mechanism of choosing [*s*_min_, *s*_max_] guarantees that λ(x) is 1:1 onto and hence invertible.

### 2.3. Intensity transformation

**Figure 3:**
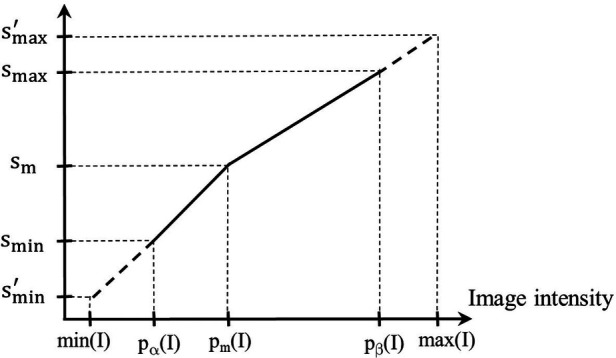
Illustration of the first standardization mapping. Input intensities in [*p_α_*(*I*), *p_m_*(*I*)] and [min(*I*), *p_α_*(*I*)) are mapped to intermediate standardized intensities by a single linear mapping. Input intensities in [*p_m_*(*I*), *p_β_*(*I*)] and (*p_β_*(*I*), max(*I*)] are mapped to intermediate standardized intensities.

A given input test image *I* ∈ *I_t_* is converted to a standardized image *I_s_* = *ψ*(*I*) by using two mappings. The first, denoted by *η*(*I*), maps input intensity *I*(*v*) at voxel *v* to output intensity *J_s_*(*v*) at *v*, where *J_s_* = *η*(*I*), on an intermediate standardized scale. The second mapping, denoted by $\lambda^{-1}(x)$, represents the inverse of the scaling transformation λ(x) mentioned above in Section 2.2 (iii). We will use Figure 3 to illustrate the first step. Overall, *η* is non-linear or piece-wise linear with two linear segments: [*p_α_*(*I*), *p_m_*(*I*)] mapped to [*s*_min_, *s_m_*]; and [*p_m_*(*I*), *p_β_*(I)] mapped to [*s_m_*, *s*_max_]. The first linear segment is extended (extrapolated) to map any input intensities *I*(*v*) in the half-open interval [min(*I*), *p_α_*(*I*)) to. In our case, since min(*I*) = *p_α_*(*I*), this (half-open) interval is empty, and thus, $s_{\min}^{\prime }=s_{\min}$. Similarly, the second segment is extended to map any input intensities *I*(*v*) in *p_β_*(I), max(*I*)] to $\left(s_{\max},\;s_{\max}^{\prime }\right]$. In our case, this half-open interval covers most of the high-uptake (and outlier) intensities in the upper tail of the histogram of *I*. Note here that the actual value of $s_{\max}^{\prime }$ is defined by the slope of the second linear segment and the actual maximum value max(*I*) in *I*. Thus, $s_{\max}^{\prime }$ will vary from image to image or patient to patient. More importantly, all intensities in I are retained faithfully and mapped to *I_s_* appropriately so that in the lower parts of the scale below *p_β_*(*I*) corresponding to mostly normal uptake values, standardized numeric meaning is achieved.

The second mapping via $\lambda^{-1}(x)$ is performed after the above transformation process. This is for bringing the transformed PET values and SUVs on the intermediate standardized scale back to their original physical meaning. The $\lambda^{-1}$ transformation is obtained as follows. Note that since *p_α_*(*I*) = 0 = *s*_min_, *λ*(*x*) is simply a scale (multiplication) factor. Since each *I* in $I_{c}$ may give rise to a different scale factor, we find $\lambda^{-1}(x)$ by first removing scale factors that are greater than one standard deviation away from the mean scale factor and then finding the mean of the remaining scale factors. $\lambda^{-1}(x)$ then is simply the reciprocal of the found mean. The overall standardization mapping *ψ*(*I*) is thus a composition^4^ of *η* and $\lambda^{-1}$.

In summary, the proposed method of standardization consists of a one-time calibration step and a transformation step that is applied to any given image. The latter step does not require any segmentation mask. In the calibration step, key parameters of the standardization mapping are estimated from a given set of PET image data sets of normal subjects. There are no parameters in the method that need manual or *ad hoc* adjustment, and the process is fully automatic once calibration is set up.

### 2.4. Iterative strategies

The method described above can be applied iteratively. That is, the standardization method can be applied to the already standardized images repeatedly. Referring to a single application of the method as applied to AC PET and SUV PET images by s-AC and s-SUV, respectively, in the iterative strategy, we form sequences of operations such as: s-AC → s-AC; s-SUV → s-SUV; s-AC → SUV; s-AC → SUV → s-SUV; etc. For example, s-AC → SUV → s-SUV connotes standardizing AC, followed by SUV estimation via Equation 1, followed by SUV standardization. For the second application of standardization, the calibration set $I_{c}$ is standardized to create set $I_{c}^{s}$ and the calibration parameters are re-estimated based on $I_{c}^{s}$.

### 2.5. Evaluation metrics

Our test set $I_{t}$ consists of two cohorts of images—a set $I_{n}$ of images of normal subjects and another set $I_{r}$ of images of non-normal subjects where repeated scans were available within 7 days of each other. For $I_{n}$, our goal is to investigate how the mean intensity within certain objects *O* varies among all images in *I_n_* before and after standardization. We expect the *coefficient of variation* of this mean intensity after standardization to be significantly lower than that before standardization since the subjects are considered normal^5^. For $I_{r}$, our goal is to assess the difference in mean intensity within *O* between the two repeat scans for each subject. We expect this difference to be significantly lower after standardization than before standardization. For both evaluation strategies, the objects considered are liver and spleen for the reasons explained in Section 2.2.

For the set of images $I_{n}$ and object *O*, we denote the *coefficient of variation* of the mean intensity *μ_O_*(*I*) within *O* over the images *I* in $I_{n}$ by CV*_O_*($I_{n}$):

(5) $$CV_{O}(I_{n})=\frac{S_{O}(I_{n})}{M_{O}(I_{n})},$$

where *M_O_*($I_{n}$) and *S_O_*($I_{n}$) are the mean and standard deviation, respectively, of *μ_O_*(*I*) over all $I\in I_{n}$. Denoting the set of standardized images corresponding to *I_n_* by $I_{n}^{s}$, our hypothesis is that $CV_{O}(I_{n}^{s})$ will be significantly lower than CV*_O_*($I_{n}$) for both AC PET and SUV PET images.

For the set of images $I_{r}$ and object *O*, we define the *mean absolute difference* MD*_O_*(*I_r_*) between the mean intensity *μ_O_*(*I*_1_) within O of the first scan image $I_{1}\in I_{r}$ of a subject and the mean intensity *μ_O_*(*I*_2_) within *O* in the second repeated scan image $I_{2}\in I_{r}$ of the same subject by:

(6) $$MD_{O}(I_{r})=\frac{1}{\left|I_{r}\right|}\sum_{I_{1},I_{2}\in I_{r}}\frac{\left|\mu_{O}(I_{1})-\mu_{O}(I_{2})\right|}{[\mu_{O}(I_{1})+\mu_{O}(I_{2})]/2}.$$

MD*_O_* expresses the average of the normalized differences between *μ_O_*(*I*_1_) and *μ_O_*(*I*_2_) over all corresponding pairs of images in $I_{r}$. We hypothesize that $MD_{O}(I_{r}^{s})$ will be significantly lower than MD*_O_*($I_{r}$), where $I_{r}^{s}$ denotes the set of standardized images corresponding to $I_{r}$, for both AC PET and SUV PET images.

## 3. Data sets, experiments, and results

### 3.1. Data sets

This retrospective study was conducted following approval from the Institutional Review Board at the Hospital of the University of Pennsylvania along with a Health Insurance Portability and Accountability Act waiver. The following data sets were utilized for this study. Our data set I contains a total of 84 FDG-PET/CT scans with the following division of the scans among subsets: |$I_{c}$| = 23; |$I_{t}$| = 61; |$I_{n}$| = 15; and |$I_{r}$| = 46; note that $I_{t}$ is a union of $I_{n}$ and $I_{r}$.

Normal scan data sets ($I_{c}$ ∪ $I_{n}$): This set includes 38 whole-body (skull vertex to toes) or near whole-body (skull base to proximal thighs) PET/CT scans with normal-appearing livers and spleens on the PET images and otherwise radiologically near-normal appearance of other organs of the body with exception of minor incidental abnormalities such as small liver cysts and lung nodules as verified by a board-certified radiologist (co-author Torigian). The scans were acquired on two different brands of scanners; they were obtained in 17 women (mean age 69, range 52–85 years, mean weight 73 kg, range 49–98 kg, mean BMI 28.14 kg/m^2^, range 17.27–38.28 kg/m^2^) previously scanned on a Biograph mCT scanner (Siemens Healthcare, Erlangen, Germany) and 21 men (mean age 44, range 30–50 years, mean BMI 26.80 kg/m^2^, range 20.80–35.10 kg/m^2^) previously scanned on a Gemini TF scanner (Philips Center, Amsterdam, The Netherlands). These 38 scans were acquired approximately 60 min after administration of approximately 15 mCi of FDG. This set is considered as the normal data set and is employed for calibration ($I_{c}$) and testing ($I_{n}$).

Repeated scan data set ($I_{r}$): This data set includes a pair of repeated near whole-body (skull base to proximal thighs) PET/CT scans from 12 men and 11 women (mean age 59, range 40–71 years) with advanced stage non-small cell lung carcinoma (mean SUV_max_ 13.61 and range 5.80–55.10 for initial scans, mean SUV_max_ 13.85 and range 4.70–55.10 for initial scans for repeat scans). The data sets were acquired on three brands of scanners: Gemini TF (Philips Center, Amsterdam, The Netherlands), Discovery LS and Discovery STE 16 slices (General Electric Healthcare, Waukesha, WI), and Biograph 40 Truepoint (Siemens Healthcare, Erlangen, Germany) PET/CT scanners as part of a prospective multicenter research study ACRIN 6678 (see Acknowledgements). All patients had previously undergone initial and repeat FDG-PET/CT imaging within 7 days without intervening therapy where repeat scans were performed using FDG administration and image acquisition parameters similar to those in the initial scans. Both initial and repeat scans had been acquired with FDG uptake delay times within 10–15 min of each other.

### 3.2. Experiments and results

#### 3.2.1. Quantitative evaluation

For quantitative evaluation, we have conducted four experiments: (E1) for comparing coefficient of variation before and after standardization on normal data sets, (E2) for comparing mean absolute difference obtained before and after standardization on repeat scans, (E3) for comparing among iterative strategies, and (E4) for comparing performance on normal data sets obtained from different brands of scanners. For experiments E1 and E2, we also included other methods commonly used in the literature (30), called Gaussian normalization and *Z*-score normalization methods as well as the original MRI standardization method (19, 20). We will refer to the Gaussian and *Z*-score methods correspondingly by G-AC, G-SUV, Z-AC, and Z-SUV, and to the MRI standardization methodology by M-AC and M-SUV.

For E1, utilizing data set $I_{n}$, we compare the coefficient of variation CV*_O_*(*I_n_*) for *O* ∈ {liver, spleen} before standardization with $CV_{O}(I_{n}^{s})$ obtained after standardization. For E2, utilizing data set $I_{r}$, we compare mean absolute difference MD*_O_*($I_{r}$) before standardization with $MD_{O}(I_{r}^{s})$ obtained after standardization. The G- and Z-methods require an estimate of the mean $\mu_{O}^{G}(I)$ and standard deviation $\sigma_{O}^{G}(I)$ of intensities within a reference organ O in each test image *I*, and hence a segmentation of *O* in each *I*, as such, we estimated $\mu_{O}^{G}(I)$ and $\sigma_{O}^{G}(I)$ for each image *I* in $I_{t}$. Further, they normalize intensities only within *O*. The Gaussian method “normalizes” intensities in a test image *I* (AC or SUV) in $I_{t}$ by dividing the voxel value *I*(*v*) by the standard deviation $\sigma_{O}^{G}(I)$. The normalized image is given by:

(7) $$I_{s}(v)=\frac{I(v)}{\sigma_{O}^{G}(I)}.$$

The normalized image in the *Z*-method is given by:

(8) $$I_{s}(v)=\frac{I(v)-\mu_{O}^{G}(I_{c})}{\sigma_{O}^{G}(I)}.$$

In Table 1, we summarize our results from the two experiments E1 and E2 by listing CV*_O_* and MD*_O_* values before standardization and for the four methods after standardization for both AC PET and SUV PET images. In the table, I^*s*^ represents the set of standardized images corresponding to I (I_*n*_ or I_*r*_) output by each of the different methods.

**Table 1:** CV*_O_* and MD*_O_* values (%) for liver and spleen derived from data set *I_n_* and *I_r_* respectively before standardization (*I*) and for the three methods after standardization (I_*s*_) for both AC PET and SUV PET images.

Metric	Organ	AC (I)	G-AC (I_*s*_)	Z-AC (I_*s*_)	SUV(I)	G-SUV (I_*s*_)	Z-SUV (I_*s*_)	M-AC (I_*s*_)	M-SUV (I_*s*_)	s-AC (I_*s*_)	s-SUV (I_*s*_)
CV*_O_*	Liver	42.28	27.29	21.62	30.10	27.28	21.51	32.23	32.52	11.48	11.66
	Spleen	37.50	20.23	17.73	27.56	20.26	17.83	31.43	33.42	12.21	12.36
MD*_O_*	Liver	9.34	10.34	14.94	8.17	10.43	15.91	25.87	27.34	3.38	4.83
	Spleen	20.82	27.91	18.35	20.13	27.5	19.13	30.76	33.23	5.04	6.26

We make the following observations from this table: (i) The proposed standardization method reduces CV significantly—by a factor of 3–4. Not surprisingly, the reductions are similar for AC PET and SUV PET images and for both organs; (ii) Although the concept underlying SUV reduces variability somewhat (by about 10% for both organs), significant residual variability remains; (iii) Compared to the mechanism underlying just standardization via SUV, the G- and Z-methods achieve slightly better harmonization of AC PET images, the Z-method performing slightly better, but they both fail to improve beyond this level for SUV images. More importantly, note that these methods require a segmentation of *O* in each test image, and standardization (normalization) is applicable only within the region of *O* and not on the whole image; (iv) Compared to the original MRI standardization method, the proposed method reduces both CV*_O_* and MD*_O_* for both organs by a factor of 3–8. This is exactly for the key reasons mentioned in Section 1, justifying the need for a new method to handle AC and SUV non-standardness; (v) The proposed standardization strategy significantly outperforms both G- and Z-methods in harmonizing both AC PET and SUV PET images; (vi) The variability seen in repeat scans in the spleen is greater than that in the liver, which is what is observed in clinical practice. Interestingly, AC PET and SUV PET images show similar variability in repeat scans; and (vii) Again, the proposed method outperforms the other methods and achieves a significant reduction in variations between repeat scans, with a residual variation of 3%–6%.

In experiment E3, utilizing metrics CV*_O_* and MD*_O_*, we compared the following iterative and the above non-iterative strategies: s-AC → s-AC; s-AC → SUV; s-AC → s-AC → SUV; s-SUV → s-SUV. The results are summarized in Table 2 for both liver and spleen. We make several key observations: (i) The SUVs resulting from s-AC → SUV are far less harmonized than directly standardizing SUV PET images (s-SUV; see Table 1). However, they are slightly more harmonized than the original SUVs (4th column in Table 1). Although s-AC achieves substantial harmonization (see Table 1), subsequently the process of estimating SUVs from the standardized AC PET images itself introduces its own non-standardness; and (ii) Repeated application of standardization (to AC PET and SUV PET images) does not seem to help since most non-standardness seems to be mitigated in the first application of standardization.

**Table 2:** CV*_O_* values (%) and MD*_O_* values (%) for liver and spleen derived from data sets *I_n_* and *I_r_*, respectively, for comparing different iterative strategies.

	s-AC → s-AC		s-AC → SUV		s-AC → s-AC → SUV		s-SUV → s-SUV	
	$CV_{O}(I_{n}^{s})$	$MD_{O}(I_{r}^{s})$	$CV_{O}(I_{n}^{s})$	$MD_{O}(I_{r}^{s})$	$CV_{O}(I_{n}^{s})$	$MD_{O}(I_{r}^{s})$	$CV_{O}(I_{n}^{s})$	$MD_{O}(I_{r}^{s})$
Liver	11.88	5.01	26.15	6.47	28.24	7.11	11.95	5.08
Spleen	12.58	6.53	25.09	9.01	27.28	11.41	13.44	7.01

For experiment E4, our goal was to study how effective the s-AC and s-SUV methods are in standardizing data sets coming from different brands of scanner. Ideally, we would like to have a sufficient number of studies in *I_r_* such that, for each subject, the repeated scans *I*_1_ and *I*_2_ of the same subject come from two different brands of scanners. Unfortunately, this is not the case, and so for E4, we chose data set $I_{n}$ where we have 17 healthy women scanned on Siemens Biograph mCT scanner and 21 healthy men scanned on Philips Gemini TF scanner. We will refer to these two subsets by $I_{n}$_1_ and $I_{n}$_2_, respectively. For this assessment, we will assume that, upon standardization, similar SUVs are expected for the same organ in $I_{n}$_1_ and $I_{n}$_2_ since the subjects are normal. We conducted two experiments, one using a subset of $I_{n}$_1_ as set $I_{c}$ for calibration and another using a subset of $I_{n}$_2_ as $I_{c}$. In the first case, let *ϑ_n_*_1_ = *I_n_*_1_ − *I_c_* and $J_{n1}^{s}$ denote the standardized version of *ϑ_n_*_1_. Using the notations related to Equation 5, we then compare the mean $M_{O}(J_{n1}^{s})$ and standard deviation $S_{O}(J_{n1}^{s})$ of the mean intensities *μ_O_*(*I*) within O over the images *I* in *ϑ_n_*_1_ with the corresponding mean and standard deviation $M_{O}{(I}_{n2}^{s})$ and standard deviation $S_{O}(I_{n2}^{s})$ of the mean intensities *μ_O_*(*I*) within *O* over the images *I* in $I_{n2}^{s}$. We expect the mean intensities $M_{O}(J_{n1}^{s})$ and $M_{O}(I_{n2}^{s})$ to be statistically indistinguishable under a *t*-test. In the second case, the setup is similar, with the roles of $I_{n}$_1_ and $I_{n}$_2_ interchanged. Table 3 summarizes the results from the two cases for liver and spleen for s-AC and s-SUV. In each case, we used 7 studies as set $I_{c}$ for calibration. From the *P*-values listed, it is clear that the mean intensities obtained after standardization using the two strategies for the two brands of scanners are statistically indistinguishable for both s-AC and s-SUV. For comparison, we also estimated the mean and standard deviation of raw SUVs of the two sets of scans $I_{n}$_1_ and $I_{n}$_2_. They were found to be *M_O_*($I_{n}$_1_) = 1.11, S*_O_*($I_{n}$_1_) = 0.50, and M*_O_*($I_{n}$_2_) = 0.83, *S_O_*($I_{n}$_2_) = 0.22, with a *P* = 0.03 for their *t*-test comparison. This, combined with the results shown in earlier tables, demonstrates that the standardization strategies overcome not only inter-subject variations in AC values and SUVs but also mitigate inter-scanner variations.

**Table 3:** *M_o_* and *S_O_* values for liver and spleen derived from data sets $J_{n1}^{s}$, $I_{n2}^{s}$, $I_{n1}^{s}$, and $J_{n2}^{s}$ for comparing different strategies based on data sets from different brands of scanners. *P*-values of statistical comparisons are also shown.

	s-AC			s-AC			s-SUV			s-SUV		
	$M_{o}(J_{n1}^{s})$	$M_{O}(I_{n2}^{s})$	*P*	$M_{O}(I_{n1}^{s})$	$M_{o}(J_{n2}^{s})$	*P*	$M_{o}(J_{n1}^{s})$	$M_{O}(I_{n2}^{s})$	*P*	$M_{O}(I_{n1}^{s})$	$M_{o}(J_{n2}^{s})$	*P*
	$S_{O}(J_{n1}^{s})$	$S_{O}(I_{n2}^{s})$		$S_{O}(I_{n1}^{s})$	$S_{O}(J_{n2}^{s})$		$S_{O}(J_{n1}^{s})$	$S_{O}(I_{n2}^{s})$		$S_{O}(I_{n1}^{s})$	$S_{O}(J_{n2}^{s})$	
Liver	4.83	4.77	0.73	4.94	4.85	0.62	4.74	4.71	0.83	4.86	4.79	0.70
	0.61	0.47		0.63	0.48		0.52	0.48		0.53	0.49	
Spleen	4.32	4.26	0.70	4.42	4.33	0.63	4.26	4.20	0.74	4.35	4.28	0.67
	0.61	0.46		0.63	0.47		0.56	0.45		0.57	0.46	

#### 3.2.2. Qualitative evaluation

**Figure 4:**
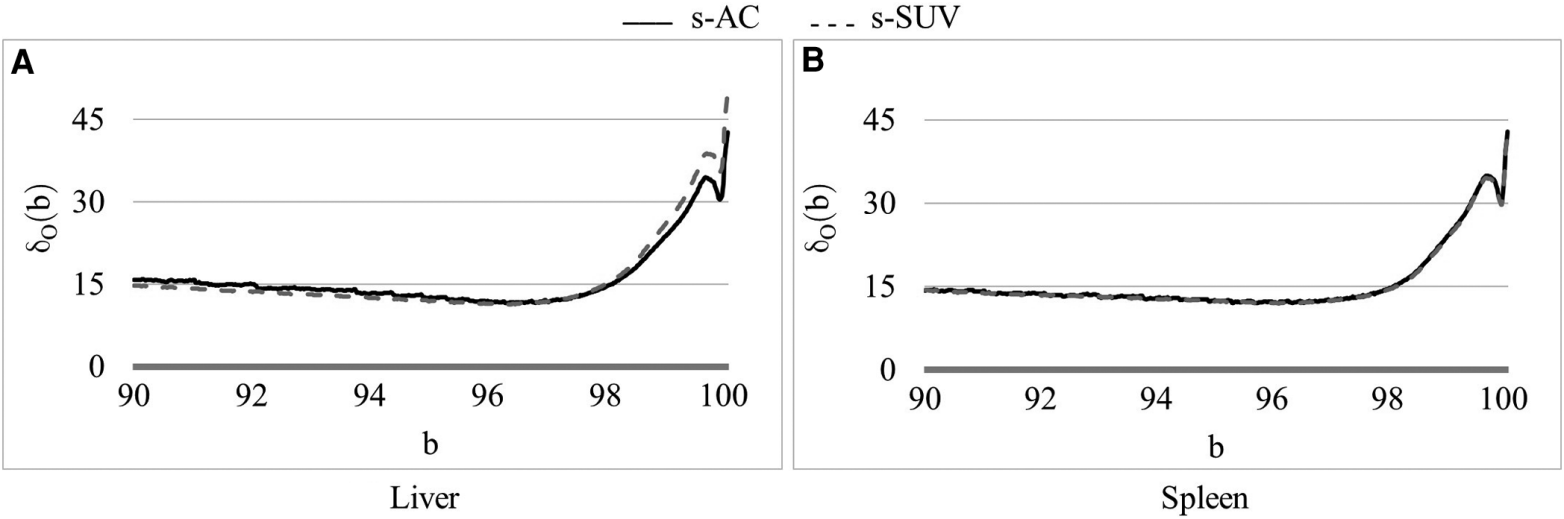
Coefficient of variation *δ*_0_(*b*) (%) as a function of the percentile value *b* in [90, 100] for both AC PET and SUV PET images for (**A**) liver and (**B**) spleen. The optimum values of b found are *β* = 96.5 for PET images.

**Figure 5:**
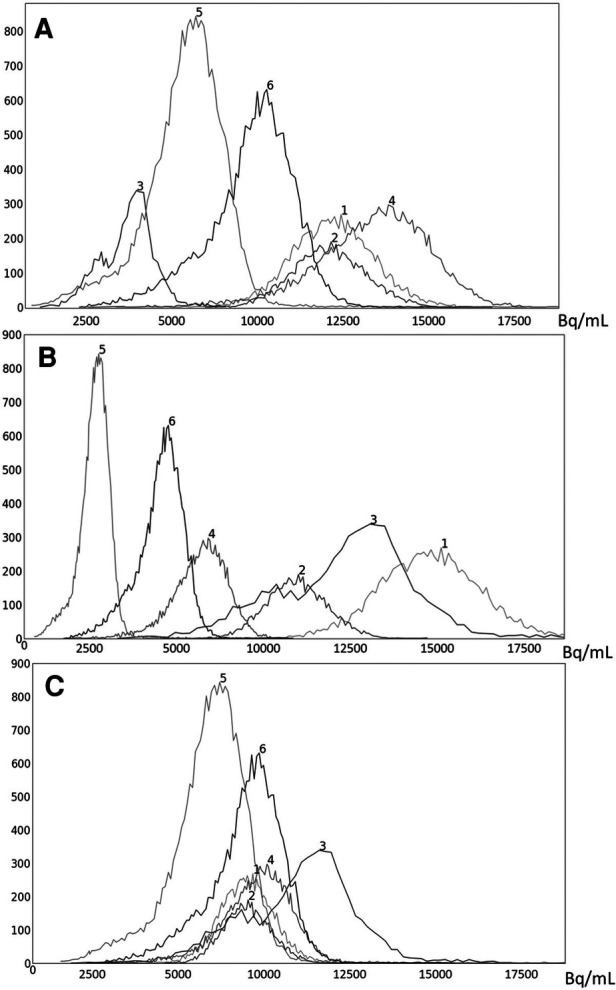
Histograms of liver 3D AC PET images of 6 subjects randomly chosen from the PET data set I*_n_*: (**A**) original AC PET image; (**B**) AC PET image scaled by linear mapping guided by the maximum value; (**C**) s-AC.

**Figure 6:**
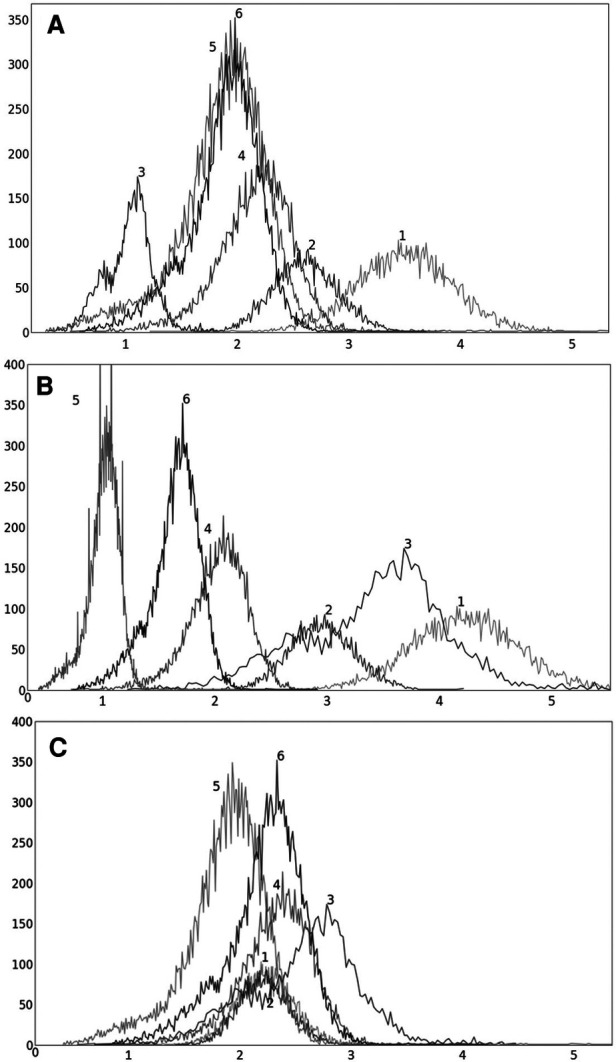
Histograms of liver 3D SUV PET images of 6 subjects randomly chosen from the SUV data set I*_n_*: (**A**) original SUV PET image; (**B**) SUV PET image scaled by linear mapping guided by the maximum value; (**C**) s-AC.

**Figure 7:**
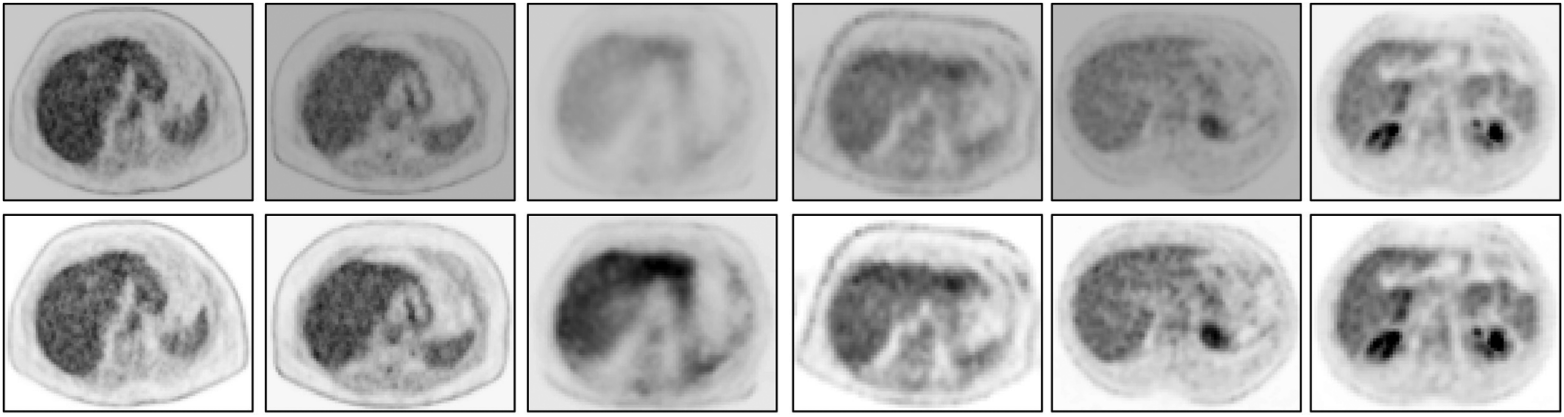
An abdominal SUV PET image slice selected from each of 6 3D body torso images *I* in $I_{n}$ before standardization (top row) and after standardization (bottom row). In each row, the images are displayed at a fixed gray map adjusted to the first image in the row.

To illustrate the performance of the s-AC and s-SUV standardization methods qualitatively, we display several graphs and images in Figures 4–7.

In Figure 4, we show the plot of *δ_O_*(*b*) (Equation 2) as a function of the upper percentile variable b for each of liver (Figure 4A) and spleen (Figure 4B) taken as a reference object. For AC standardization, the optimal values *β* found for *b* with [*b_L_*, *b_H_*] = [90, 100] for liver and spleen were identical, namely, *β* = 96.4. Similarly, for SUV standardization, these values were identical, with *β* = 95.6. As seen in Figure 4, the coefficient of variation suddenly rises for *b* > ∼98, suggesting a cut off percentile beyond which image intensities are extremely variable from subject to subject.

To illustrate the uniformization effect of our standardization strategy, we display histograms of the liver AC PET images selected from $I_{n}$ from 6 subjects in Figure 5 as follows: (a) before standardization, (b) after linear mapping determined by the maximum value in the image, and (c) upon standardization (s-AC). In Figure 6, we display histograms from SUV PET images from the liver of the same subjects where the layout is similar to that of Figure 5. The purpose of (*b*) is to demonstrate that just a linear mapping of the entire AC/SUV range to a common scale does not help to standardize, and that standardization of the whole image requires a non-linear mapping. In fact, linear mapping makes matters worse—the histograms are more spread out after mapping. The point made in Table 1 about the SUV estimation process taking care of some non-standardness, but not all, existing in AC PET images is borne out in Figures 5A, 6A. The histograms of subjects 5 and 6, which were far apart in AC PET images, come close together in SUV PET images. However, for other subjects, such a mitigation of non-standardness did not take place.

Finally, we demonstrate via image slice display at fixed gray map windows how uniformity of numeric meaning is achieved after standardization. Figure 7 displays (top row) an abdominal slice selected from each of the 6 SUV data sets in $I_{n}$. The same slices from the same data sets after standardization are also displayed (bottom row). For each row, a fixed gray map window is used which is adjusted optimally for the first image in the row. It can be readily seen that standardization facilitates the use of fixed gray map windows, whereas fixed windows do not offer optimum slice visualization prior to standardization owing to non-standardness of intensity meaning.

As alluded to in Section 2, parameter *s*_max_ was chosen to be 5.00 for SUV PET images so as to not lose intensity in normal portions of the activity. The logic behind this selection is as follows. We require that, for any two distinct SUV values *x* and *x* + *dx* in *I*, we will be able to differentiate between them with a difference of at least *dx*. We assumed *dx* = 0.01 since, in clinical practice, this level of discriminability is adequate. By examining all SUV PET images that we analyzed and the associated standardization mappings, we found that *s*_max_ ≥ 4.53 fulfills this requirement for *dx* = 0.01. Therefore, we set *s*_max_ = 5.00. Similarly, for AC PET, we set *s*_max_ = 50,000 Bq/ml.

For the calibration data set $I_{c}$ of AC PET images, we observed that the scale factor λ(x) ranged from 2.66 to 13.49 The mean scale factor after leaving out extreme values was 6.00, and thus, $\lambda^{-1}$ as a multiplication factor was 1/6.00 = 0.167. For the calibration data set $I_{c}$ of SUV PET images, we observed that the scale factor λ(x) ranged from 1.66 to 5.94 The mean scale factor after leaving out extreme values was 2.50, and thus, $\lambda^{-1}$ as a multiplication factor was 1/2.50 = 0.405.

## 4. Discussion and conclusion

We proposed a new methodology for standardizing AC PET and SUV PET images individually, called s-AC and s-SUV, respectively, to overcome the effect of undesired factors that impede accurate quantitative analysis for clinical and research purposes. The methods can be directly applied to AC/SUV PET images without requiring the parameters related to the scanner, image acquisition, or the patient. They consist of a one-time calibration step wherein the parameters pertaining to the standardization mapping are estimated once and for all using a reference organ. This is followed by the transformation step wherein any given image is subjected to the standardization mapping. The methods are fully automatic, requiring no per-image interactive input or adjustment of parameters. Moreover, both s-AC and s-SUV preserve the original meaning of activity concentration and Standardized Uptake Value. Their effectiveness in significantly improving the tissue-specific AC/SUV numeric meaning is demonstrated quantitatively using scan data from four different scanners via two metrics: (i) reduced variability in the scans of normal subjects within liver and spleen and (ii) improved reproducibility of image intensities within these organs in repeated scans of patients with different pathologies. Improvement in uniformization is also demonstrated qualitatively through displays of histograms and images at fixed gray map settings.

The proposed s-AC and s-SUV methods have been evaluated in comparison with two commonly used strategies, namely, Gaussian and *Z*-score intensity normalization, demonstrating the following key advantages: (i) s-AC and s-SUV significantly outperform the G- and Z-methods in terms of the above quantitative metrics. The latter methods do not seem to be able to go beyond the normalization achieved by the SUV process and leave considerable residual non-standardness; (ii) G- and Z-methods perform normalization only within the organ of interest and not on the whole image and require a pre-segmentation of the organ of focus. In contradistinction, the proposed methods standardize the whole image and do not require segmentation of any organs or the reference organ to be within the field of view of the scan. The only segmentation, not precise but rough, required is that of the entire body region which can be performed quite effectively by thresholding as demonstrated in the paper; and (iii) Since the proposed methods standardize the whole image, they can be employed as a pre-processing step to facilitate further analysis of the images for image segmentation, disease quantification, response assessment, etc.

Although the optimal value *β* for the upper percentile was 96.4 for AC and 95.6 for SUV, the behavior of the *δ_O_*(*b*) function was almost identical for AC PET and SUV PET images (Figure 4). Given this and the observation that, at the optimal value, there is no sharp valley in *δ_O_*(*b*), we surmise that setting *β* = 96.0 would not make much difference in the effectiveness of standardization in terms of metrics CV*_O_* and MD*_O_*. This indeed turned out to be true, with *β* = 96.0, the new metric values becoming, for liver in s-AC: $CV_{O}(I_{n}^{s})\;\;=\;11.47$, $MD_{O}(I_{r}^{s})\;=\;3.38$; and for liver in SUV: $CV_{O}(I_{n}^{s})\;\;=\;11.67$, $MD_{O}(I_{r}^{s})\;=\;4.84$. The absolute maximum difference is less than 0.01% of the previous metric values, as can be seen by comparing these new values with the entries in Table 1. For spleen as well, the difference turned to be less than 0.01% with *β* = 96.0. Another interesting finding is that direct standardization of SUV PET images is better than standardizing AC PET images followed by converting them to SUV images (see Table 2). Our recommendation is that if AC PET images are needed for subsequent image processing/analysis operations, then perform s-AC processing, and if SUV images are the end goal, then perform s-SUV processing. Also, as shown in Table 2, one application of the standardization mapping takes care of the underlying non-standardness in AC/SUV PET images and there is no benefit in repeated application.

We used liver and spleen separately as a reference organ. For FDG-PET imaging, the optimum values *β* obtained for both organs are similar. If some other object or tissue region is used as reference, the optimum value *β* needs to be estimated via Equation 3 by using data set $I_{c}$ in the calibration step. Similarly, if one does PET imaging with radiotracers other than FDG, then the liver and spleen may not necessarily be the best choice since the accumulation and distribution of radiotracer uptake may differ from that of FDG, and therefore, the estimation of *β* may have to be redone for each individual type of radiotracer utilized. Although performed on a small sample, our analysis indicates that the standardization mapping can mitigate variations potentially coming from different brands of scanners.

One limitation of this work is the rather small number of cases utilized in testing method performance, especially as related to inter-scanner variation of SUVs. Although our existing data sets came from multiple scanners, we did not have a sufficiently large number of studies from each of several brands of scanners. One of our future goals is to acquire such data sets and test our method's ability to standardize both AC PET and SUV PET intensities across all major brands of scanners currently used in clinical practice.

In summary, the proposed s-AC and s-SUV algorithms involve a one-time calibration step which requires a set $I_{c}$ of FDG-PET data sets of normal subjects and the segmentation mask of a reference organ or tissue region for each image. All parameters needed by the method are then estimated automatically by the algorithms. Subsequently, any given FDG-PET image of a patient can be *standardized* automatically by using the parameters estimated in the calibration step. The algorithms are easy to implement and computationally inexpensive. Their ability to drastically reduce variations inherent in the existing SUV measurement process, especially as evidenced by our repeated scan experiments, suggests that the s-SUV measures may be used for disease measurement highly reliably.

## Data Availability

The original contributions presented in the study are included in the article/Supplementary Material, further inquiries can be directed to the corresponding author.

## Footnotes

^1^Many papers have been published on the subject of intensity standardization subsequent to the introduction of the concept by Nyul et al. in the name of intensity *normalization* or *harmonization* (20, 21). Normalization implies uniform (or linear) scaling of a variable, whereas the process under consideration involves non-linear mappings. Similarly, harmonization implies making image value meaning uniform without reference to a specific absolute standard value scale, whereas AC PET and SUV have standard meaning. Therefore, we suggest that standardization is a more appropriate term to describe the process.

^2^It is not necessary for the whole image to be normal or disease-free. As we explain in Section 2.2, our method uses a reference organ such as liver. “Normal” implies that the reference organ should be normal in all images in the set I*_c_*.

^3^Our intent is to reduce the variation in image intensity values in “normal” tissues of the same type across subjects as much as possible while leaving the natural variations that exist unaltered.

^4^There is a slight abuse of notation here. *η* maps an image *I* into another image *J_s_*. Equivalently, it can also be thought of as mapping an input voxel value *I*(*v*) to another value assigned to the same voxel in the output, namely *J_s_*(*v*). Since λ and $\lambda^{-1}$ map voxel values to voxel values, with the voxel mapping interpretation of *η*, composition of *η* and $\lambda^{-1}$ makes sense.

^5^Here “normal” means the entire liver and spleen are radiologically normal on the PET images and that the remainder of the image is radiologically near-normal with exception of minor incidental abnormalities such as small liver cysts or lung nodules.
